# A Prospective Analysis of Health Information Portals in Four Years

**DOI:** 10.3390/ijerph17134761

**Published:** 2020-07-02

**Authors:** Hanna Choi, Soo-Kyoung Lee

**Affiliations:** 1 Department of Nursing Science, Nambu University, Gwangju 62271, Korea; hanna.choi.kr@gmail.com; 2 College of Nursing, Keimyung University, Daegu 42601, Korea

**Keywords:** eHealth, health information, consumer, portal, internet

## Abstract

*Objectives:* We examined 17 health information portals to determine the status of web-based health information services in the United States (USA), South Korea, the United Kingdom (UK), and Australia. *Methods:* We analyzed longitudinal trends in 35 items of online health information over four years among representative health information portals (eight based in the USA, seven in South Korea, one in the UK, and one in Australia), focusing on external portal structure, content scope, service characteristic, and service function with four stakeholder groups of six stakeholders. *Results:* The most notable change was in the service items, and overall, in 44.1% of total items: 17.6% in service characteristic, 41.2% in external portal structure, 58.8% in service function, and 58.8% in content scope change. More specifically, these changes included increases in the “mobile application utility” (service function), “use of personal health records” on public health portals (content scope change), “Charts and videos” (service characteristic), and “renewal date” (external portal structure). *Conclusions:* This review of existing health portals will be a footnote for enabling health care providers to confirm whether the needs of consumers are reflected on their website with high reliability. Furthermore, these findings will help to enhance the quality of portals by delivering relevant information to stakeholders and to the consumers of online health information.

## 1. Introduction

With the current coronavirus outbreak, countless fake health-related news articles are being published to gain people’s attention. Such fake news amplifies anxiety and creates confusion. Therefore, the need for proper health information is greater than ever before.

There were several discussions regarding the need for proper health-related information which existed around the year 2000, when the interest of online health information increased due to the emergence of high-speed internet for consumers [1,2,3]. Furthermore, even though current health-related portals existed before 2000, it is still hard to find reliable online health information debates and research. In South Korea, the latest study analyzing the status of online health information was published around a decade ago [4,5].

The internet usage rate reached 82.1% in South Korea, and the number of internet users is constantly increasing [1]. In other words, there are more active consumers who search for and select health information independently [2]. Furthermore, they expect the information they acquire to be constantly updated with information which meets their expectations. 

However, it is difficult to conclude whether these portals actually provide the latest high-quality information. Numerous health-related portals undergo rapid changes with new portals constantly being launched, going defuncted, and being renewed. Health information has a major impact on consumers’ daily lives such as on their communication with medical professionals, healthcare decision making, the exploration of health and disease information for themselves, families, and friends, online purchasing of drugs or vitamins, search for good doctors, or when making appointments at hospitals and clinics [3]. The high reliability on online health-related information from the current consumers suggests that it is necessary to engage in a continuous and systematic analysis of web-based health information through analyzing the content and service characteristics. While there is a more related study that investigated the meaning and role of the Korean National Health Information Portal [6] and a study by the Korea Institute for Health and Social Affairs that surveyed the usage of online healthcare services [7], neither proposes a direction for the services based on a systematic analysis. Unfortunately, the studies that have systematically analyzed web-based information are lacking abroad as well. Thankfully, various analyses are being conducted based on large-scale and periodic surveys of health information services and consumers’ usage of such information, such as the Health Information National Trend Survey [8,9]. Therefore, this study investigated the status of online health information and comprehensively analyzed the contents and services provided by some of the most popular online health information portals in the United States, South Korea, the United Kingdom, and Australia. More specifically, this study analyzed the structures of health information portals, the scope and contents of the information, the features of the services provided, and the functions of providing those services. We ultimately aimed to not only promote the provision of effective health information, but also help online health information consumers become “smart consumers”, who can effectively discern and utilize the given information in their decision making by relating to observations made in four years from 2014 to 2018, years which saw important changes in health information portals.

## 2. Method

### 2.1. Study Design

This was a study that longitudinally analyzed the health information services provided by 17 popular health information portals.

### 2.2. Instruments

This study attempted to the cover external and internal portals to evaluate all aspects. One of the external aspects of a portal includes external structure measurement, or in other words, developing a sitemap. Other traits include the internal aspects of a portal—evaluating the content scope, what and how to provide services (service characteristic).

(1) We analyzed the external portal structures using Song’s (2006) tool, which classifies the structure into a sitemap, feedback, and date of update [5].
(2) The content scope of the provided information was analyzed by choosing 17 services commonly provided by each site and organizing them into a matrix for analysis, based on a 15-category classification method created by five health information experts. These categories included: (1) disease cause, (2) symptoms/signs, (3) diagnosis, (4) test/care, (5) treatment, (6) patient experiences (frequently asked questions), (7) drug/drug side effects, (8) disability/ rehabilitation, (9) hospital and clinic/nursing institutions, (10) cost of care/ insurance, (11) personal health management (e.g., exercise, nutrition, postoperative management), (12) preventative educational materials, (13) customer service, (14) personal health records, and (15) lifecycle-related content.
(3) The service characteristic of each portal was analyzed using seven items devised by Cho and Woo [10]. The items were as follows: (1) whether the links connecting to the other reference pages or portals are valid, (2) whether demanded health information is provided sufficiently on each page, (3) whether the provided health-related information is easily comprehensible, (4) whether ways to ask for help when practicing or using provided health information is specified or a question-answer service is provided, (5) whether figures, diagrams, and videos are frequently used to help readers understand, (6) whether an internal search tool is available to search for needed information, and (7) whether precautions to take when using the provided health information are specified.
(4) The functions of providing different services across portals (service function) were analyzed using the classification by Son (2006), according to which service are classified into eight types. Remote care was excluded, as it is only partially permitted in South Korea, and the availability of mobile services was added as a category in response to the popularization of mobile services. In addition, other services commonly provided by web portals, such as relevant news and symptom finders, were included. Thus, a total of 10 items were analyzed: (1) categorized health information, (2) online health consultations, (3) link service, (4) appointment, (5) advertisement, (6) community, (7) online product sales, (8) mobile service, (9) health-related news, and 10) symptom finder [5].

### 2.3. Data Collection and Analysis

On the portals, four stakeholder groups—6 people, (1) the developer, (2) researchers, (3) third parties, and (4) end users (consumers)—decided upon inclusion and exclusion criteria by consensus. The stakeholders selected 17 representative portals among the major technology-leading countries for inclusion. Minor blogs, private websites, and social networking services (SNSs) were excluded.

The 17 popular health portals were run by private and public health insurance providers, including government agencies, other public agencies, the Healthcare Management Organization (HMO), and the Accountable Care Organization (ACO), as well as other private businesses (Table 1). Portals of national public health insurance, private health insurance, and popular private portals in South Korea, the United States, the United Kingdom, and Australia were ultimately included. Portals that primarily focused on services for specific groups of individuals or diseases were excluded. The 17 health information portals consisted of four public portals [11,12,13,14] and three private portals in South Korea [14,15,16,17], three public portals [18,19,20] and five private portals in the United States (U.S.A.) [21,22,23,24,25], and one public portal each in Australia [26] and the United Kingdom (U.K.) [27].

The 17 services were chosen based upon: (1) if possible, a country that speaks English as a mother-tongue since there will be limitations in translation when it is not from an English-spoken country, (2) it should be from a country that is highly developed in ICT technology as these countries will have an advanced implementation of the information, (3) comprehensively, have different cultures since examples of different races should be included, in this way Korea was chosen as it is from an Asian culture and ranked in first place for ICT development. In addition, the author can fully understand the information provided, (4) the ratio of public and private service providers were to be similar as comparing differences in content aspects will also be useful, and (5) some services were chosen based upon their connection with health insurance to examine the differences in services that are and that are not connected.

In 2013, the World Health Assembly has recognized the need for health data standardization to be part of eHealth systems and services. Therefore, to analyze the degree of standardization in health data, including the eHealth system and service system, this research was first started in 2014. In 2016, the WHO Executive Board stated, “mHealth: use of mobile wireless technologies for public health,” reflecting the increasing importance of this resource for health services delivery and public health, given their ease of use, and emphasized broad reach and wide acceptance. To examine the degree of effect that mHealth has towards portal platforms in health information usage as discussed in 2017, by the WHO, this study was restarted [28].

The content scope and services as of September 2014 were structurally analyzed. Then, one professor and one medical information expert reviewed whether the online health information content and services changed in January 2018 to examine the process of change and the evolution of web-based health information. 

## 3. Results

### 3.1. Comparison of Portals between Private and Private Organization

Depending on the organization that provides the information, the portals were divided as private or public (Table 2). Overall, in both 2014 and 2018, private health information portals tend to provide more services than public ones. Specifically, in both 2014 and 2018, public health portals tend to provide better services in external portal structure, however those of internal status—content scope, service characteristics, and service function—were better provided by private health information portals than those of public.

### 3.2. External Portal Structure

When compared to 2014, in 2018 we observed changes in 41.2% (seven portals) of the portals. In 2014, 76.5% (*n* = 13) and 94.1% (*n* = 16) of portals provided a sitemap and feedback, but only 23.5% (four portals) of the portals provided a date of update. In 2018, these percentages increased to 88.2% (15 portals), 100.0% (17 portals), and 47.1% (eight portals), respectively (Table 1).

Moreover, 64.7% (33 items) of the table was marked True (T) if the webpage provided the service fully, 33.3% (17 items) were marked as False (F) if the service was not provided at all, and 2.0% (one item) was marked Unclear (U) if undefined.

### 3.3. Content Scope

We observed changes in 17.6% (three portals) of the content scope over the four years. The percentage of portals that provided information on hospitals or clinic/nursing institutions increased from 82.4% (14 portals) to 94.1% (16 portals), and the number of portals providing information on personal health records increased from 41.2% (seven portals) to 47.1% (eight portals). With the exception of some portals—the American public health insurance schemes Medicare and Medicaid, Korea’s National Health Insurance Review and Assessment Service, and the National Health Insurance Service—all the portals provided information on disease causes, symptoms and signs, diagnosis, testing/care, treatment, and preventative educational materials. Furthermore, 82.4% (14 portals) of the services provided information on patient experiences and hospital and clinic/nursing institutions, while 76.5% (13 portals) of the services provided information on cost of care and insurance. A total of 64.7% (11 portals) of portals provided information on personal health management and drugs’ side effects, while 58.8% (10 portals) provided customer service-related content. A total of 35.3% (six portals) of the services provided information on lifecycle-related content, while 23.5% (four portals) provided information on disability and rehabilitation-related content (Table 2 and Table 3). 

Furthermore, 66.7% (169 items) of the table was marked True (T) if the webpage has provided the service, 33.7% (85 items) were marked F if the service was not provided at all, and 0.4% (1 item) was marked Unclear (U) if undefined.

### 3.4. Service Characteristic 

Table 4 shows the service characteristic of the health information. We observed a change in 23.5% (four portals) over the four years, with the largest change being the percentage of services providing diagrams and videos increasing from 47.1% (eight portals) to 64.7% (11 portals). 70.6% (12 portals) of the portals had “strongly agree” ratings for “valid connection to reference links,” “sufficiency of information,” “accuracy and comprehensibility of content,” and “search feature.” Most portals were assessed to have “moderate” ratings for “possibility to ask for help” and “diagrams, figures, videos.” For “warnings,” 70.6% (12 portals) of portals received “moderate” ratings, and 29.4% (five portals) received “strongly disagree” ratings. Finally, for “precautions for using contents,” 29.4% (five portals) received “strongly disagree” ratings and 70.6% (12 portals) received “moderate” ratings.

Moreover, 60.7% (72 items) of the table was marked True (T) if the webpage had provided the service fully, 32.7% (39 items) were marked U if only a section of the page had the service, and 6.7% (eight items) were marked False (F) if the service was not provided at all.

### 3.5. Service Function

Over four years, we observed changes in 64.7% (11 portals) of the service delivery characteristics across the portals. The changes were observed were mostly in portals targeting the general public (64.7%, 11 portals), followed by portals targeting the insured (23.5%, four portals) and portals targeting patients and their families (11.8%, two portals). Furthermore, 41.2% of the portals (seven portals) provided mobile services in 2014, while in 2018, all portals did (Table 5). About 82.4% (14 portals) of the portals provided health information-related news, symptoms, and links. 

Moreover, 48.8% (83 items) of the table was marked True (T) if the webpage provided the service fully, 51.2% (87 items) were marked False (F) if the service was not provided at all (Table 6 and Table 7).

## 4. Discussion

This study analyzed the health information portals to promote consumer health by helping them better utilize health information. With the exception of those from the studies by Song [5] and Woo [10], there are no studies comparing portals providing health information in South Korea and abroad. Furthermore, while there is a 17-item scale called DISCERN that is used to assess the quality and reliability of health information per se, it cannot be used to evaluate the portals that provide that information [29]. Therefore, we longitudinally analyzed the characteristics of services and content provided by 17 popular public and private health portals in South Korea and abroad, and confirmed the changes in the service characteristics, external portal structures, service function, and content scope over four years. The findings of this study provide us with a systematic understanding of the changing web environments from multiple viewpoints, specifically by allowing us to confirm the features of services provided by each web portal and how they differ by target consumers and the purpose of the portal.

Regarding the structural characteristics of the portals, most Korean portals provided feedback, while only four portals overall provided a date of update. This suggests that web portals should do more to ensure the provision of reliable and up-to-date information. We also noted no marked changes in newspaper articles over the course of four years, apart from the addition of the date of publication. There are some concerns about whether the date of update means the final date of revision or the date of posting, or, for publications, the date of publication [30]. Knowing the date of update is not only a basic need for consumers to access up-to-date information but also a fundamental factor of providing health information; thus, more care should be taken on this matter. One feature that distinguished the U.K.’s National Health Service (NHS) portal was that it presents the date of initial posting as well as the dates of updates.

Regarding the content scope, the public health portals changed their services by allowing members who have logged on to the portal to download their personal health records. This indicates the increased use of personal health records. Currently, consumers can decide the scope of personal health records they wish to download, although the exact information will vary according to the service provider. However, this is meaningful in that consumers can now verify and manage at least a portion of their health information [31]. 

Broadly speaking, the portals operated by public health insurance companies focused on providing information about health insurance and relevant policies and regulations, while 65% (11 portals), including those operated by other public agencies, focused on providing disease-related content, patient experiences, information on hospitals or clinic/nursing institutions, the cost of care and insurance products, personal health management, and drugs/drug side effects. Only 58.8% (10 portals) of the portals provided customer service-related content, and often only to registered members. Personal information-related issues might be attributable to the fact that there are challenges in the representativeness of services provided by the government and the official opinions presented by public agencies. 

Regarding the service provision functions, 41.2% (seven portals) of the portals offered mobile services in 2014, whereas all portals provided mobile services in 2018. This indicates a drastic change in the use of mobile applications for delivering health information and shows that the health industry has actively accepted and capitalized on the popularity of mobile applications and services [31]. 

One service function that distinguished the public health portals from private portals was that the former did not tend to provide services relating to competitive or financial components such as appointment services, advertisements, and online product sales. In addition, private portals actively utilized the customer service feature to promote arresting contents such as cosmetic surgery and sex. Hidoc was the only portal that offered a quoting service for cosmetic surgery and special deals as a separate menu. Kormedi.com connected consumers to hospitals, enabling consumers to search for and rate popular surgeons and engage in online consultations with physicians. On the other hand, although HealthiN deleted disability and rehabilitation-related information as part of their update in 2014, in order to ensure that their information was up to date, the National Health Information Portal provided a link to the content of the National Rehabilitation Center website for the same reason. Whereas public health portals included comprehensive contents for the general public and socially disadvantaged groups, as opposed to simply providing eye-catching content, private portals actively reflected the latest trends in response to consumer demands. For example, one foreign private portal, MedlinePlus, offered information about the health of caregivers and pets, which suggests that it had a better understanding of the demands of cultural consumers compared to public portals [20]. 

As for the service characteristic, all 17 portals tended to offer valid connections to reference links which was sufficient, accurate, and easily comprehensible information, and search functions. However, most portals did not specify precautions for the provided content. Thus, it might be useful for portals to provide precautions to help consumers better utilize the given information appropriately and prevent the misuse of such information. Korean private portals stood out not only for their recommendation of nearby hospitals but also for their offering direct, though limited, responses from physicians through the portals. By contrast, the U.K.’s NHS portal stood out in terms of allowing its users to revisit health issues of interest based on previous search records once they log on to the portal. It also featured portals that might be of interest to specific users.

Even though the portals might all be in the range of a health information portal, there are differences in the strength and weakness in the provided service based upon the service provider. For instance, if the portal was provided by a public provider, the portals tend to focus on outer structure rather than contents or services. Furthermore, we were able to notice differences in the service platform due to the emerging development of internet and mobile conditions. The analysis of portal development over four years will be helpful for both public and private circumstances in defining or guiding a direction for providing information in health information portals.

The interpretation of our findings should be made with caution, as the selected portals do not represent the entirety of health portals, and such portals are continually changing at a rapid pace. Nevertheless, our findings are useful as a reference for developing a standard for health portals based on its specific purpose. Furthermore, it will help promote these portals in order to help online health information consumers manage their health based on the latest web-based health information.

However, the distributions of portals providing advertisements, communities, online product sales, appointment services, and online health consultation services differed according to the natures of the portals. The fact that we excluded portals that provided online health consultation services only to registered members might have contributed to these results. In fact, even though all the portals provided health information, their specific purposes and objectives would differ according to their target population. Therefore, it might be impossible to define such portals using one or two standards. Nevertheless, it is possible to understand the information currently provided to consumers and develop measures on how to utilize such information according to the specific information provider by analyzing these portals.

## 5. Conclusions

This study was conducted from 2014 to early 2018 with the evolution time of these portals, therefore a further analysis of portals after 2018 is required, including the evolutionary changes in SNSs, mobile utility, and its universalization as the channel to provide new convergent e-services. Moreover, it is dangerous to make quick generalization from this study as there are four portals within Korea. To gain generalization of the research results, more portals from various nations will be required. If there is an analysis based upon more countries’ health information portal, it would better represent the trend of health information provided by portals.

## Figures and Tables

**Table 1:** Characteristics of the selected studies.

	Portal	Country	Population	Outcome					
				Public, Private	Health Insurance	External Portal Structure	Content Scope	Service Characteristic	Service Function
1	National Health Insurance Service (NHIS) [11]	South Korea	General	Public	True	2 (66.7)	5 (33.3)	2 (28.6)	3 (30.0)
2	Health Insurance Review Agency (HIRA) [12]	South Korea	General	Public	True	3 (100.0)	6 (40.0)	2 (28.6)	5 (50.0)
3	HealthiN [13]	South Korea	General	Public	False	3 (100.0)	13 (86.7)	4 (57.1)	6 (60.0)
4	National Health Information Portal (NHIP) [14]	South Korea	General	Public	False	3 (100.0)	14 (93.3)	4 (57.1)	6 (60.0)
5	Naver Health [15]	South Korea	General	Private	False	3 (100.0)	11 (73.3)	4 (57.1)	7 (70.0)
6	HIDOC [16]	South Korea	General	Private	False	3 (100.0)	13 (86.7)	6 (85.7)	10 (100.0)
7	kormedi.com [17]	South Korea	General	Private	False	2 (66.7)	12 (80.0)	6 (85.7)	8 (80.0)
8	Medicare [18]	U.S.A.	Insurer	Public	True	2 (66.7)	3 (20.0)	0	3 (30.0)
9	Medicaid [19]	U.S.A.	Patients and families	Public	True	2 (66.7)	2 (13.3)	1 (14.3)	2 (20.0)
10	BlueCross BlueShield [21]	U.S.A.	Insurer	Private	True	2 (66.7)	5 (33.3)	2 (28.6)	5 (50.0)
11	Kaiser Permanente [22]	U.S.A.	Insurer	Private	True	2 (66.7)	10 (66.7)	5 (71.4)	9 (90.0)
12	Aetna [23]	U.S.A.	Insurer	Private	True	2 (66.7)	7 (46.7)	6 (85.7)	5 (50.0)
13	Healthdirect [26]	Australia	General	Public	False	1 (33.3)	13 (86.7)	5 (71.4)	4 (40.0)
14	NHS Choices [27]	U.K.	General	Public	False	3 (100.0)	14 (93.3)	6 (85.7)	4 (40.0)
15	MedlinePlus [20]	U.S.A.	General	Public	False	3 (100.0)	10 (66.7)	5 (71.4)	5 (50.0)
16	WebMD [24]	U.S.A.	General	Private	False	2 (66.7)	10 (66.7)	4 (57.1)	7 (70.0)
17	Mayo Clinic [25]	U.S.A.	Patients and families	Private	False	2 (66.7)	13 (86.7)	5 (71.4)	7 (70.0)

**Table 2:** Comparison of private and public in 2014 and 2018 (frequency, %).

Year	Range	Organization	Total	External Portal Structure	Content Scope	Service Characteristic	Service Function
2014	True	Public	152 (42.8)	18 (54.4)	74 (44.0)	29 (40.8)	31 (37.3)
		Private	203 (57.2)	15 (45.5)	94 (56.0)	42 (59.2)	52 (62.7)
	Unclear	Public	28 (68.3)	1 (100.0)	1 (100.0)	26 (66.7)	-
		Private	13 (31.7)	-	-	13 (33.3)	-
	False	Public	135 (67.8)	8 (47.1)	60 (69.8)	8 (88.9)	59 (67.8)
		Private	64 (32.2)	9 (52.9)	26 (30.2)	1 (11.1)	28 (32.2)
2018	True	Public	171 (46.2)	22 (55.0)	80 (50.0)	31 (41.9)	38 (44.4)
		Private	199 (53.8)	18 (45.0)	80 (50.0)	43 (58.1)	58 (60.4)
	Unclear	Public	26 (56.5)	-		25 (67.6)	1 (100.0)
		Private	20 (43.5)	-	8 (100.0)	12 (32.4)	-
	False	Public	118 (65.9)	5 (45.5)	55 (63.2)	7 (87.5)	51 (69.9)
		Private	61 (34.1)	6 (54.5)	32 (36.8)	1 (12.5)	22 (30.1)

**Table 3:** Changed external portal structure of health information portals. (Frequency, %).

Service	Site Map	Feedback	Date of Update	2014	2018
NHIS	T	T	F	2 (66.7)	2 (66.7)
HIRA	T	T	F→T	2 (66.7)	3 (100.0)
HealthiN	T	T	U→T	2 (66.7)	3 (100.0)
NHIP	T	T	T	3 (100.0)	3 (100.0)
Naver Health	F→T	F→T	F→T	0	3 (100.0)
HIDOC	T	T	T	3 (100.0)	3 (100.0)
kormedi.com	F	T	T	2 (66.7)	2 (66.7)
Medicare	F→T	T	F	1 (33.3)	2 (66.7)
Medicaid	T	T	F	2 (66.7)	2 (66.7)
BlueCross BlueShield	T	T	F	2 (66.7)	2 (66.7)
Kaiser Permanente	T	T	F	2 (66.7)	2 (66.7)
Aetna	T	T	F	2 (66.7)	2 (66.7)
Healthdirect	F	T	F	1 (33.3)	1 (33.3)
NHS Choices	T	T	F→T	2 (66.7)	3 (100.0)
MedlinePlus	T	T	T	3 (100.0)	3 (100.0)
WebMD	T	T	F	2 (66.7)	2 (66.7)
Mayo Clinic	T	T	F	2 (66.7)	2 (66.7)
2014	13 (76.5)	16 (94.1)	4 (23.5)		
2018	15 (88.2)	17 (100.0)	8 (47.1)		

T: True, U: Unclear, F: False.

**Table 4:** Changes in the content scope on health information portals 1 (frequency, %).

Service	Disease/Cause	Sing/Symptom	Diagnosis	Inspection/Treatment	Treatment	Patient Experience (FAQ *)	Medication/Medication Side Effect	Disability/Rehabilitation	2014	2018
NHIS	F	F	F	F	F	T	F	F	1 (12.5)	1 (12.5)
HIRA	F	F	F	F	F	T	T	F	2 (25.0)	2 (25.0)
HealthiN	T	T	T	T	T	T	T	F	7 (87.5)	7 (87.5)
NHIP	T	T	T	T	T	T	T	T	8 (100.0)	8 (100.0)
Naver Health	T	T	T	T	T	T	T	F	7 (87.5)	7 (87.5)
HIDOC	T	T	T	T	T	T	T	F	7 (87.5)	7 (87.5)
kormedi.com	T	T	T	T	T	T	F	F	6 (75.0)	6 (75.0)
Medicare	F	F	F	F	F	F	F	F	0	0
Medicaid	F	F	F	F	F	F	F	F	0	0
BlueCross BlueShield	T→F	T→F	T→F	T→F	T→F	T	F	F	6 (75.0)	1 (12.5)
Kaiser Permanente	T	T	T	T	T	F	T→F	F	6 (75.0)	5 (62.5)
Aetna	T→U	T→U	T→U	T→U	T→U	T→U	T→U	T→U	8 (100.0)	0
Healthdirect	T	T	T	T	T	T	F→T	F	6 (75.0)	7 (87.5)
NHS Choices	T	T	T	T	T	T	T	T	8 (100.0)	8 (100.0)
MedlinePlus	T	T	T	T	T	T	T	F	7 (87.5)	7 (87.5)
WebMD	T	T	T	T	T	T	T	F	7 (87.5)	7 (87.5)
Mayo Clinic	T	T	T	T	T	T	T	T	8 (100.0)	8 (100.0)
2014	13 (76.5)	13 (76.5)	13 (76.5)	13 (76.5)	13 (76.5)	14 (82.4)	11 (64.7)	4 (23.5)		
2018	11 (64.7)	11 (64.7)	11 (64.7)	11 (64.7)	11 (64.7)	13 (76.5)	10 (58.8)	3 (17.6)		

T: True, U: Unclear, F: False. * FAQ (Frequently Asked Questions).

**Table 5:** Changes in the content scope on health information portals 2 (frequency, %).

Service	Clinic/Medical Institution	Medical Expense/Insurance	Personal Health Care	Education Materials/Prevention	Customer Consultation	Personal Health Record	Life Cycle Management	2014	2018
NHIS	T	T	F	F	T	T	F	4 (57.1)	4 (57.1)
HIRA	T	T	F	F	T	T	F	4 (57.1)	4 (57.1)
HealthiN	T	F	T	T	U→T	T	T	5 (71.4)	6 (85.7)
NHIP	T	T	T	T	T	F	F→T	5 (71.4)	6 (85.7)
Naver Health	F→T	T	T→F	T	F	T→F	T	5 (71.4)	4 (57.1)
HYDAC	T	F→T	T	T	T	F	T	5 (71.4)	6 (85.7)
kormedi.com	T	T	T	T	T	F	T	6 (85.7)	6 (85.7)
Medicare	T	T	F	F	F	T	F	3 (42.9)	3 (42.9)
Medicaid	F	T	F	F	F	T	F	2 (28.6)	2 (28.6)
BlueCross BlueShield	T	T	F	T	T	F	F	4 (57.1)	4 (57.1)
Kaiser Permanente	T	T	T	T	T	F	F	5 (71.4)	5 (71.4)
Aetna	T	T	T	T	T	F→T	T	6 (85.7)	7 (100.0)
Healthdirect	F→T	T	T	T	F	F→T	T	4 (57.1)	6 (85.7)
NHS Choices	T	T	T	T	T	F	F→T	5 (71.4)	6 (85.7)
MedlinePlus	T	F	T	T	F	F	F	3 (42.9)	3 (42.9)
WebMD	T	F	T	T	F	F	F	3 (42.9)	3 (42.9)
Mayo Clinic	T→F	T	T	T	T	T	F	6 (85.7)	5 (71.4)
2014	14 (82.4)	13 (76.5)	12 (70.6)	13 (76.5)	10 (58.8)	7 (41.2)	6 (35.3)		
2018	16 (94.1)	14 (82.4)	11 (64.7)	13 (76.5)	11 (64.7)	8 (47.1)	8 (47.1)		

T: True, U: Unclear, F: False.

**Table 6:** Changes in the service characteristics of health information portals (frequency, %).

Service	Connect Reference Link	Sufficient Amount	Understandable Content	Request Help	Charts, Videos	Search	Precautions	2014	2018
NHIS	T	U	U	T	F→U	U	F	2 (28.6)	2 (28.6)
HIRA	T	U	U	T	F	U	F	2 (28.6)	2 (28.6)
HealthiN	T	T	T	U	T	T	U	4 (57.1)	4 (57.1)
NHIP	T	T	T	U	T	T	U	4 (57.1)	4 (57.1)
Naver Health	T	T	T	U	T	T	U	4 (57.1)	4 (57.1)
HIDOC	T	T	T	T	T	T	U	6 (85.7)	6 (85.7)
kormedi.com	T	T	T	T	T	T	U	6 (85.7)	6 (85.7)
Medicare	U	U	U	U	F	U	F	0	0
Medicaid	T	U	U	U	F	U	F	1 (14.3)	1 (14.3)
BlueCross BlueShield	T	T	U	T	T	U	F	2 (28.6)	2 (28.6)
Kaiser Permanente	T	T	T	T	U	T	U	5 (71.4)	5 (71.4)
Aetna	T	T	T	T	U→T	T	U	5 (71.4)	6 (85.7)
Healthdirect	T	T	T	T	U	T	U	5 (71.4)	5 (71.4)
NHS Choices	T	T	T	T	U→T	T	U	5 (71.4)	6 (85.7)
MedlinePlus	T	T	T	U	U→T	T	U	4 (57.1)	5 (71.4)
WebMD	T	T	T	U	T	T	U	4 (57.1)	4 (57.1)
Mayo Clinic	T	T	T	T	T	T	U	5 (71.4)	5 (71.4)
2014	16 (94.1)	13 (76.5)	12 (70.6)	10 (58.8)	8 (47.1)	12 (70.6)	0		
2018	16 (94.1)	13 (76.5)	12 (70.6)	10 (58.8)	11 (64.7)	12 (70.6)	0		

T: True, U: Unclear, F: False.

**Table 7:** Changed service function of the health information portals. (Frequency, %).

Service	Health Information	Health Consultation	Link	Appointment	Advertising	Community	Sale of Goods	Mobile Service	Health Related News	Find Symptoms	2014	2018
NHIS	F	F	T	F	F	F	F	T	T	F	3 (30.0)	3 (30.0)
HIRA	F→T	F	T	F	F	F→T	F	F→T	T	F	2 (20.0)	5 (50.0)
HealthiN	T	F→T	T	F	F	F	F	T	T	T	5 (50.0)	6 (60.0)
NHIP	T	F	T	F	F	F→T	F	T	T	T	5 (50.0)	6 (60.0)
Naver Health	T	F→T	T	F	T	F	F	T	T	T	6 (60.0)	7 (70.0)
HIDOC	T	T	T	F→T	T	T	T	T	T	T	9 (90.0)	10 (100.0)
kormedi.com	T	T	T	F	T	T	F	F→T	T	T	7 (70.0)	8 (80.0)
Medicare	F	F	T	F	F	F	F	F→T	T	F	2 (20.0)	3 (30.0)
Medicaid	F	F	T	F	F	F	F	F→T	F	F	1 (10.0)	2 (20.0)
BlueCross BlueShield	T→F	F	T	F	T	F	T	F→T	T	F	5 (50.0)	5 (50.0)
Kaiser Permanente	T	F	T	T	T	T	T	F→T	T	T	8 (80.0)	9 (90.0)
Aetna	T	F	T	F	T	F	T	F→T	F	F	4 (40.0)	5 (50.0)
Healthdirect	T	F	T	F	F	F→U	F	F→T	T	T→F	4 (40.0)	4 (40.0)
NHS Choices	T	F	T	F	F	F	F	F	T	T	4 (40.0)	4 (40.0)
MedlinePlus	T	F	T	F	F	F	F	T	T	T	5 (50.0)	5 (50.0)
WebMD	T	F	T	F	T	T	F	T	T	T	7 (70.0)	7 (70.0)
Mayo Clinic	T	F	T	T	T	F	F	F→T	T	T	6 (60.0)	7 (70.0)
2014	13 (76.5)	2 (11.8)	17 (100.0)	2 (11.8)	8 (47.1)	4 (24.5)	4 (24.5)	7 (41.2)	15 (88.2)	11 (64.7)		
2018	13 (76.5)	4 (24.5)	17 (100.0)	3 (17.6)	8 (47.1)	6 (35.3)	4 (24.5)	16 (94.1)	15 (88.2)	10 (58.8)		

T: True, U: Unclear, F: False.
